# Novel Antimicrobial Composites Modified with Nanosilver, CuSO_4_, Benzethonium Chloride, and ZnO

**DOI:** 10.3390/ma19143053

**Published:** 2026-07-15

**Authors:** Karolina Kiełczewska-Klim, Beata Podkościelna, Katarzyna Szałapata, Monika Osińska-Jaroszuk, Vladyslav Vivcharenko, Magdalena Jaszek

**Affiliations:** 1 Department of Polymer Chemistry, Maria Curie-Sklodowska University in Lublin, Gliniana 33, 20-614 Lublin, Poland; 2 Department of Biochemistry and Biotechnology, Maria Curie-Sklodowska University in Lublin, Akademicka 19, 20-033 Lublin, Poland; katarzyna.szalapata@mail.umcs.pl (K.S.); monika.osinska-jaroszuk@mail.umcs.pl (M.O.-J.); magdalena.jaszek@mail.umcs.pl (M.J.); 3 Department of Tissue Engineering and Regenerative Medicine, Medical University of Lublin, Chodzki 1, 20-093 Lublin, Poland; vladyslav.vivcharenko@umlub.edu.pl

**Keywords:** antimicrobial properties, biofilm formation, cytotoxicity, bactericidal and fungicidal activity, modified cross-linked methacrylate polymer materials, special additives

## Abstract

The antibiotic and drug resistance of various bacterial and fungal strains poses a significant challenge to medicine and industry. The subject of numerous studies is how to limit the spread of microorganisms and biofilm formation on various surfaces. This research focuses on the antibacterial and antifungal properties of cross-linked methacrylate-based composites for specific applications. These composites were modified using 10 wt.% of compounds with scientifically proven antimicrobial properties. These include nanosilver, copper sulphate, benzethonium chloride, and zinc oxide. The antimicrobial potential against the following bacteria and fungi was determined: Gram-positive bacteria (*Staphylococcus aureus*); Gram-negative bacteria (*Pseudomonas aeruginosa* and *Escherichia coli*); and the pathogenic fungi *Candida albicans* and *Aspergillus niger*. Using the modified disc-diffusion method alongside a serial dilution method demonstrated an inhibitory effect on the viability and formation of bacterial and fungal biofilms. It was demonstrated that—in liquid cultures—composites containing benzethonium chloride inhibited the growth of *P. aeruginosa* by over 75%, more than 50% of *E. coli* and more than 70% of *S. aureus*. Growth inhibition of *C. albicans* exceeded 80% for selected composites (BPA.DM + NVP + CuSO_4_, BPA.DM + NVP + ZnO), while all composites inhibited the growth of *A. niger* by more than 45%, and in some cases (BPA.DM + HEMA + CuSO_4_, BPA.DM + HEMA + Ag, BPA.DM + MMA + Ag and BPA.DM + AEH + CuSO_4_) by more than 90%. Additionally, these composites significantly reduced biofilm formation on their surfaces. Modification with zinc oxide and benzethonium chloride resulted in materials that were non-toxic to normal human skin fibroblasts. To sum up the obtained results, it can be stated that these multifunctional materials with antibacterial properties could be used in medical devices, coatings, and other specialised applications where microbial contamination is a significant issue.

## 1. Introduction

A significant problem that occurs in public, industrial, and medical facilities is the high risk of microbial contamination of surfaces that are frequently touched by users. Commonly used methods of disinfecting such surfaces involve the use of chemicals. It should be noted that such agents do not always prevent the adhesion of microorganisms to surfaces, as well as the formation of bacterial or fungal biofilm between cleaning cycles. One strategy for addressing this problem involves the development of antimicrobial materials that will be able to provide passive and continuous protection of the surfaces they cover [1,2,3].

Polymer composites are easily processable and durable. For this purpose, they are a particularly interesting group of materials with practical properties. The addition of modifiers with antimicrobial properties to the polymer matrix enables the design of a material that can be used as a coating for countertops, work surfaces, and other surfaces that are exposed to frequent contact with users. Recent years have seen intensive development in polymer chemistry. This enables the design of composites containing inorganic, organic, and bio-inspired additives, which are characterized by different mechanisms of antimicrobial activity [4,5,6].

An interesting group of modifiers are metal nanoparticles, especially copper, zinc oxide, and silver. Their mechanism of activity is based on the release of metal ions that interact with the cell membranes of microorganisms. They disrupt enzymatic activity and enable the formation of reactive oxygen species. Nanoparticles can also interact directly with the cell membrane of bacteria, resulting in damage to their structure and increased permeability [7,8,9,10]. The size of nanoparticles and their degree of dispersion in the polymer matrix are significant in their application. To ensure prolonged antimicrobial activity while limiting cytotoxicity, the kinetics of ion release must also be controlled [11,12].

Another group includes additives that exhibit contact activity. These contain quaternary ammonium salts, silane surface modifiers, and cationic polymers. Their mechanism of action is based on electrostatic interaction between positively charged functional groups present in the material and negatively charged cell membranes of microorganisms. Direct contact destabilizes the membrane and causes cell lysis. The advantage of using such additives is their long-lasting antimicrobial effect without significant release of biocide into the environment. However, it should be noted that the effectiveness of these substances may be limited when the surface is heavily soiled [13,14,15].

Bio-inspired additives are also increasingly being applied. These include chitosan and peptide-polymer systems. These materials have a positive biocompatibility profile. Their mechanism of action is based on electrostatic attraction to the cell membranes of microorganisms. This leads to their destabilization and sometimes also to disturbances in the metabolic processes taking place in the cell. It is also possible to combine bio-derived polymers with inorganic nanoadditives, which allows for high antimicrobial efficacy and maintains safety in use [16,17,18,19].

Although significant progress has been made in recent years in the development of composite materials with antimicrobial properties, scientists still face several technological and application challenges. It is difficult to ensure long-lasting antimicrobial activity under conditions of mechanical wear, to limit the migration of additives, and to reduce potential toxicity. It is also important to prevent the development of microbial resistance. Therefore, current technologies focus on combining different antimicrobial strategies to obtain durable, safe, and effective materials [20,21,22].

Despite numerous reports on methacrylate composites with antimicrobial properties, most studies have focused on materials based on a single polymer matrix or containing a single antimicrobial agent. The influence of the polymer matrix properties on the effectiveness of different antimicrobial modifiers has not yet been clearly established. Therefore, a systematic comparison of composites containing reactive diluents with different physicochemical properties, which may affect both the material structure and its biological activity, is warranted. The present study constitutes a segment of a more extensive research project that is focused on the development and comprehensive characterization of methacrylate-based composites modified with antimicrobial agents. Several studies have previously examined specific physicochemical aspects of these materials, encompassing their structural characteristics, the release behavior of active modifiers, and thermal stability [23,24,25]. The antimicrobial modifiers investigated in this study were deliberately selected to represent different mechanisms of antimicrobial action. Metallic agents (nanosilver, copper(II) sulphate, and zinc oxide) primarily exert their activity through metal ion release and oxidative stress, whereas benzethonium chloride represents a quaternary ammonium compound acting predominantly via disruption of microbial membranes. Such a selection enabled direct comparison of antimicrobial agents with distinct modes of action, providing a broader understanding of the relationship between polymer matrix composition, antimicrobial mechanism, and biological performance [26,27]. The aim of this study was to biologically evaluate methacrylate-based composites prepared using four composite formulations differing in the active diluent and modified with four antimicrobial agents representing different classes of compounds, namely nanosilver, copper(II) sulphate, zinc oxide, and benzethonium chloride. This unified experimental design enabled direct comparison of the influence of both the polymer matrix composition and the antimicrobial modifier on the antibacterial, antifungal, antibiofilm, and cytocompatibility properties of the composites.

## 2. Materials and Methods

### 2.1. Chemicals and Synthesis of Composites

BEN—benzethonium chloride was obtained from Lonzagard^®^ (Basel, Switzerland); HEMA—2-hydroxyethyl methacrylate; AEH—2-ethylhexyl acrylate; MMA—methyl methacrylate; NVP—1-vinyl-2-pyrrolidone (NVP), BPA.DM—bisphenol A glycerolate dimethacrylate (BPA.DM); Ag—nanosilver; ZnO—zinc oxide; CuSO_4_—copper(II) sulphate; IQ (Irgacure 651)—2,2-dimethoxy-2-phenylacetophenone were obtained from Sigma-Aldrich (Taufkirchen, Germany).

Methacrylate-based composites were prepared by UV polymerization, using bisphenol A glycerol dimethacrylate (BPA.DM) as the base monomer. BPA.DM was mixed with one of four reactive diluents, namely N-vinylpyrrolidone (NVP), 2-hydroxyethyl methacrylate (HEMA), methyl methacrylate (MMA) or 2-ethylhexyl acrylate (AEH), in a mass ratio of 7:3. Before adding the antimicrobial modifier, the monomer mixture was maintained at 65 °C to facilitate homogenization and degassing. Next, 10% by mass of the selected antimicrobial modifier (nanosilver, CuSO_4_, ZnO, or benzethonium chloride), calculated relative to the total mass of the monomer, was added to the monomer mixture. A photoinitiator (Irgacure) was then added, in an amount of 3% by mass of the monomer content. The preparations were thoroughly mixed, poured into glass moulds separated by Teflon spacers, and cured under UV radiation (160 W mercury lamps) for 30 min. To complete the polymerization, the resulting composites were subjected to final curing at 85 °C for 4 h. The preparation procedure was described in previous patent applications [28,29].

### 2.2. Evaluation of Antimicrobial Potential

Before starting the tests, the composite materials were cut into 1 × 1 cm squares and UV-sterilized for 10 min on each side. Overnight cultures of bacteria and fungi were prepared before each test. The following strains were used: a Gram-positive strain of *Staphylococcus aureus* (ATCC 25923), and Gram-negative strains of *Escherichia coli* (ATCC 25922) and *Pseudomonas aeruginosa* (ATCC 27853). Two fungal strains were also selected: *Candida albicans* (ATCC 10231) and *Aspergillus niger* (accession number G13). All the biological materials used in this study were obtained from the American Type Culture Collection (LGC Standards, Kiełpin, Poland).

A. Evaluation of antimicrobial potential on agar plates

A total of 20 mL of sterile Mueller–Hinton agar medium was placed on each Petri dish and left to solidify. Glucose (in an amount of 2% by weight) was added to the medium intended for testing with fungi. The *inoculum* concentration was 0.5 McFarland. A total of 200 µL of each *inoculum* was placed on agar plates and spread thoroughly over the entire surface. Fragments of composite materials containing 10 wt.% of the selected modifier were placed on the prepared plates, with each fragment being placed in three replicates. Composites that did not contain a modifier were used for reference (control samples). The plates were then placed in a laboratory incubator at 37 °C for 24 h for bacteria or 48 h for fungi. After this time, the composite fragments were removed from the plates, and the zones of growth inhibition were measured using a caliper. The plates were also scanned using a GBox (Syngene, Baltimore, MD, USA).

B. Evaluation of antimicrobial potential in liquid cultures

A total of 2.5 mL of liquid Mueller–Hinton medium (Sigma Aldrich, Taufkirchen, Germany) (the fungi medium was supplemented with glucose) was placed in sterile 12-well plates. Then, 100 µL of the appropriate overnight culture of fungi or bacteria, at a concentration of 0.5 McFarland, was added to the wells containing the culture medium. Fragments of composite materials were then placed in the appropriate wells. Each composite was prepared in three replicates. A material without the modifier additive was used as a reference (control sample). The optical density was measured using a Tecan SPARK plate reader (Spark, Tecan, Grödig, Austria) at 600 nm. Multi-well plates containing the culture medium and composite material fragments were placed in a laboratory incubator at 37 °C for 12 h for bacteria and 24 h for fungi. After this time, the optical density was measured again. For this purpose, 100 µL of fluid was taken from each culture and placed in 96-well plates. The process was repeated after 24 h for bacteria and after 48 h for fungi. Percentage growth inhibition was determined using the formula 100 − (*ODp*/*ODk*) × 100%, where *ODp* is the optical density of the test sample and *ODk* is the optical density of the control sample. The collected results were presented as mean ± standard deviation (SD) from at least three independent experiments (*n* = 3).

C. Bacteria and fungi survival rate determination

Plates from earlier described assay (subparagraph B) were used to conduct further analyses to determine the level of pathogenic microorganisms’ survival after contact with composites in liquid cultures. For this purpose, fragments of composites were carefully removed from the wells of the plate. A total of 20 µL of 1% 2,3,5-triphenyltetrazolium chloride (TTC) was added to each well of the plate and placed in a laboratory incubator for 2 h at 37 °C. After this time, the survival of bacteria and fungi was assessed. For each variant, a photo documentation was performed, showing the intensity of the insoluble red formazan, the intensity of which is directly proportional to the number of live and metabolically active microorganisms.

D. Assessment of biofilm formation

The following steps were taken to prepare sterile 12-well plates for the assessment of biofilm formation. The plates were prepared and filled with 2.5 mL of liquid Mueller–Hinton agar medium (supplemented with glucose for fungi). A total of 100 µL of 1% 2,3,5-triphenyltetrazolium chloride (TTC) was added to this mixture and samples were incubated. Fragments of composite materials that had been in contact with liquid bacteria culture for 24 h or liquid fungi culture for 48 h (samples of composites from experiment described in subparagraph B) were thoroughly rinsed in sterile distilled water to remove all non-adherent planktonic bacterial or fungal cells. Three replicates were performed for each composite. After 24 h of incubation for bacteria or 48 h for fungi in fresh medium with addition of 1% TTC solution, biofilm formation on the composite material surfaces was visually assessed. The presence of metabolically active microorganism cells results in the conversion of TTC into a red, insoluble formazan. The presence of red coloration was used to confirm biofilm formation.

### 2.3. Cytotoxicity Assessment of Composite Materials

Normal human skin fibroblasts (BJ cell line, CRL-2522™, ATCC-LGC Standards, Teddington, UK) [30] obtained from the American Type Culture Collection were employed to evaluate cytotoxicity of the tested materials. Cells were maintained in Eagle’s Minimum Essential Medium (EMEM 30-2003, ATCC-LGC Standards, Teddington, UK) supplemented with 10% (*v*/*v*) fetal bovine serum (FBS; Pan-Biotech GmbH, Aidenbach, Bavaria, Germany), 1% (*v*/*v*) streptomycin/penicillin solution (Sigma-Aldrich Chemicals, Warsaw, Poland) at 37 °C, 95% of air humidity containing 5% CO_2_. Cytotoxicity of the produced materials was assessed using the MTT test in accordance with ISO 10993-5 [31]. Accordingly, 1.5 × 10^4^ cells were seeded per well of a 96-well tissue culture-treated plate in 100 μL of EMEM and incubated for 24 h. Next, the culture medium was replaced with material extracts prepared according to ISO 10993-12 [32], and the cells were cultured further for 24 h and 48 h. After both incubation intervals, BJ cell viability was estimated using the MTT colorimetric assay according to the procedure described earlier [33]. Cell viability was calculated as a percentage of the absorbance value of the cells treated with polystyrene extract (negative control).

### 2.4. Statistical Analysis

The collected cytotoxicity results were presented as mean ± standard deviation (SD) from at least three independent experiments (*n* = 3). Statistical comparisons were conducted using one-way ANOVA followed by Dunnett’s post hoc test, with *p* < 0.05 considered significant (GraphPad Prism 8.0.0; GraphPad Software Inc., San Diego, CA, USA).

## 3. Results

The research presented in this paper focused on assessing the antibacterial and antifungal properties of new methacrylate-based composites enriched with additives with previously described antimicrobial properties. The cytotoxic activity of the synthesized components was also assessed. The first stage of the analysis presented in this paper involved the use of a modified diffusion-circulation method. Results relating to described composite materials were earlier presented in part in the monograph’s chapter [34]. Results relating to zinc oxide-modified composites and their antibacterial activity, including growth inhibition zones, growth inhibition percentage, and biofilm formation, were presented in our previous study [25]. The present work extends these investigations by evaluating the antifungal activity of the same materials against *C. albicans* and *A. niger* using analogous experimental approaches. Table 1 visualises the antibacterial activity of NVP-containing composites against bacteria. All analysed composite materials exhibited activity that inhibited bacterial growth upon contact. No bacterial growth was observed under the surface on which the composite material fragment was located. This also applies to the control composite, for which an additional growth inhibition zone was observed around the surface on which the composite was located (0.2 cm). The largest inhibition zones were observed for BPA.DM + NVP + BEN (0.6 cm for *S. aureus*, 0.4 cm for *E. coli*, and 0.3 cm for *P. aeruginosa*) and BPA.DM + NVP + Ag (0.4 cm for *P. aeruginosa*). Contact activity was observed for *E. coli* and *S. aureus* with CuSO_4_- and Ag-modified materials (no visible growth inhibition zones around the composite contact surface).

Table S1 (Supplementary Materials) presents scans of plates for composites containing AEH as an active diluent. As with NVP, all composite materials exhibited an inhibitory effect on bacterial growth when in contact with the plate. Growth inhibition zones were observed around the surface of all composite materials containing modifiers. The exception was BPA.DM + AEH + Ag for *S. aureus*. The largest zones were observed for BPA.DM + AEH + BEN (0.6 cm for *S. aureus*) and BPA.DM + AEH + Ag (0.4 cm for *E. coli*).

Bacterial growth was very effectively inhibited by composite materials containing HEMA (Table S2—Supplementary Materials). Adding BEN as a modifier significantly improved the composite’s performance compared to the control sample. Growth inhibition zones of 0.3 cm for *P. aeruginosa*, 0.2 cm for *E. coli*, and 0.7 cm for *S. aureus* were determined for the BPA.DM + HEMA + BEN composite material.

A similar trend was observed for MMA-containing composites (Table S3—Supplementary Materials). The largest zones of growth inhibition were measured for BPA.DM + MMA + BEN (0.4 cm for *P. aeruginosa*, 0.3 cm for *E. coli*, and *S. aureus*) and BPA.DM + MMA + Ag (0.3 cm for *P. aeruginosa*).

Table 2 shows scans that confirm the antifungal activity of NVP-containing composites. In the case of *A. niger*, no fungal growth was observed beneath the surface on which the composite was placed. This is relevant to both control materials and composites with the incorporation of antimicrobials. Similar results were obtained for *C. albicans*. The exception is the composite containing CuSO_4_. A few fungal colonies were visible under the surface in contact with the composite. Tables S4–S6 (Supplementary Materials) summarise the results for composites containing AEH, HEMA, and MMA. The results of the analysis were very similar. For *A. niger*, growth inhibition was visible under the surface where the composite material was located. For *C. albicans*, however, only a few fungal colonies were visible under the surface of the composite plate for CuSO_4_-modified composites. In all analysed cases, no growth inhibition zone was observed at the composite contact surface.

Table 3 and Table S7 (Supplementary Materials) summarize the results for Gram-positive and Gram-negative bacteria. Almost all of the analyzed composite materials significantly inhibited the growth of *P. aeruginosa* after 24 h of contact. The exceptions were BPA.DM + NVP + Ag, BPA.DM + AEH, BPA.DM + HEMA and BPA.DM + MMA + CuSO_4_. The highest values were obtained for BPA.DM + NVP + BEN (95.3 ± 1.7), BPA.DM + AEH + BEN (78.9 ± 2.1), and BPA.DM + MMA + BEN (88.8 ± 2.3). For *E. coli*, the degree of bacterial growth inhibition was often greater after 12 h than after 24 h. The highest values were recorded for composites containing NVP as an active diluent (53.5 ± 2.2 for BPA.DM + NVP, 57.3 ± 0.1 for BPA.DM + NVP + BEN, and 55.1 ± 1.1 for BPA.DM + NVP + Ag), as well as for BPA.DM + MMA + BEN (63.1 ± 0.9). In the case of *S. aureus*, the calculated degree of bacterial growth inhibition was greater after 24 h. In a significant number of cases, the percentage inhibition increased compared to the value calculated after 12 h. The highest values were obtained for BPA.DM + AEH + BEN (78.5 ± 0.3), BPA.DM + HEMA + BEN (85.5 ± 2.1), and BPA.DM + MMA + Ag (74.6 ± 0.4).

Table 4 and Table S8 (Supplementary Materials) present the growth inhibition values for the fungi *C. albicans* and *A. niger*. For *C. albicans*, the highest values of growth inhibition were observed after 24 h. The determined % growth inhibition was 81.4 ± 0.3 for BPA.DM + HEMA + CuSO_4_, 78.5 ± 0.3 for BPA.DM + AEH + BEN, 85.5 ± 2.1 for BPA.DM + HEMA + BEN, and 74.6 ± 0.4 for BPA.DM + MMA + Ag. DM + NVP + CuSO_4_ was 80.3 ± 0.9, and for BPA.DM + NVP + ZnO was 80.1 ± 1.4. For *A. niger*, the % growth inhibition was greater than 45% for all composite materials after 24 and 48 h. The highest values were determined for BPA.DM + AEH + CuSO_4_ (91.9 ± 1.9), BPA.DM + HEMA + CuSO_4_ (91.1 ± 1.7), BPA.DM + HEMA + Ag (95.4 ± 0.3) and BPA.DM + MMA + Ag (95.6 ± 1.7).

The insoluble red 1,3,5-triphenylformazane (TPF) is precipitated from 2,3,5-triphenyltetrazolium chloride (TTC) in the presence of metabolically active bacterial cells. The intensity of the solution’s colour is directly proportional to the number of living cells present [35]. This method was used to assess the survival rate of bacteria after contact with composite materials (Table 5 and Table S9—Supplementary Materials). For *P. aeruginosa*, the composites exhibited very good bactericidal properties, as confirmed by the absence of red coloration of the culture medium. The only exception was the BPA.DM + MMA + Ag composite material. For *E. coli*, NVP composites containing a modifier also limited bacterial growth, and the culture medium in the wells did not turn red. Similar observations were made for composites containing modifiers that included MMA and HEMA in their composition. For AEH composite materials, the addition of CuSO_4_ and Ag also significantly inhibited bacterial growth. In the case of *S. aureus*, a bacteriostatic effect was observed for AEH MMA and HEMA composite materials modified with BEN and CuSO_4_, as well as for materials without the addition of a modifier that contained NVP and HEMA.

Regarding *C. albicans*, composite materials demonstrated very good fungicidal properties, as confirmed by the absence of red discoloration of the culture medium (Table 6 and Table S10—Supplementary Materials). For *A. niger*, significant growth inhibition in relation to control was observed for BPA.DM + NVP, BPA.DM + NVP + BEN and BPA.DM + NVP + ZnO. This was also visible for BPA.DM + AEH, BPA.DM + AEH + CuSO_4_, BPA.DM + AEH + ZnO, BPA.DM + HEMA, and composites containing CuSO_4_ as a modifier or MMA as an active diluent, except for BPA.DM + MMA + Ag.

Table 7 and Table 8 and Tables S11 and S12 (Supplementary Materials) show scans of composite fragments after using the TTC method as a reaction substrate. The presence of red coloring confirms the formation of a bacterial or fungal biofilm on the surface of the tested material. For *P. aeruginosa*, red coloration was visible on the surface of the BPA.DM + AEH + BEN composite. The BPA.DM + AEH and CuSO_4_-modified composites, however, showed red coloration—only—at the edges, possibly due to these fragments having a rough surface resulting from cutting. Similar results were obtained for *E. coli*. In this case, red discoloration appeared on the surface of the BPA.DM + NVP composite. For *S. aureus*, red formazan precipitation was visible on the surfaces of the composites without a modifier, as well as on the BPA.DM + AEH + CuSO_4_ and BPA.DM + MMA + CuSO_4_ composites. Slight red discoloration was visible at the edges of the composite materials that had come into contact with *C. albicans* and *A. niger* fungi, which can again be explained by the sharp, rough edges of the material, which promote adhesion of microbial cells.

**Figure 1:**
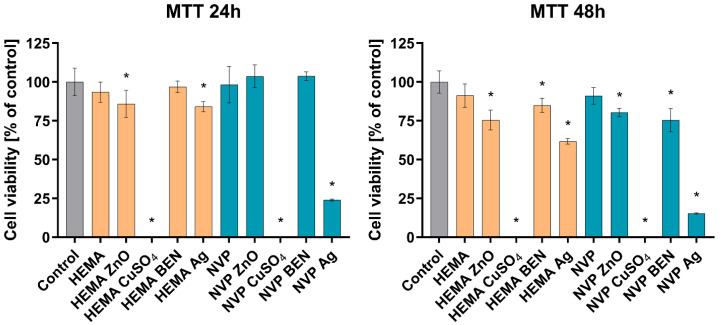
Cytotoxicity assessment of the fabricated composites against BJ cells according to ISO 10993-5 [31] standard using the MTT assay (* statistically significant results compared with negative control (*p*  <  0.05); one-way ANOVA followed by Dunnett’s test).

The conducted cytotoxicity evaluation revealed that composite materials modified with copper sulphate in both the HEMA and NVP groups were highly toxic, as the viability of the cells treated with the sample extracts was close to 0% compared to the non-toxic control. A cytotoxic effect on BJ cells was also observed in the case of samples modified with nanosilver, where, after 48 h, cell viability dropped below 70%, reaching 61% for the HEMA sample and 15% in the case of the NVP composite. The remaining samples, modified with zinc oxide and benzethonium, were non-toxic. Despite the statistically significant reduction in fibroblast viability after 48 h, the tested materials were characterized by cell viability above 75% (Figure 1).

## 4. Discussion

Numerous studies have confirmed the varying antimicrobial activity of metal nanoparticles against both Gram-negative and Gram-positive bacteria. The generation of reactive oxygen species (ROS) has been identified as the antimicrobial action mechanism of copper and copper oxide in particular. Studies based on electron paramagnetic resonance (EPR) have confirmed the formation of hydroxyl radicals as well as superoxide anion radicals. This observation suggests the potential significance of oxidative stress in the antimicrobial activity of copper-modified materials. The resultant ROS led to several deleterious effects, including protein oxidation, lipid peroxidation of cell membranes, and damage to nucleic acids. This results in disruptions to fundamental cellular metabolic processes [36]. A large number of amines and carboxyl groups have been shown on the surface of bacterial cells in the literature, as well as a high affinity of copper ions for these groups. The destabilization of the cell membrane and the consequent increase in its permeability are facilitated by the adsorption of Cu^2+^ ions onto the cell surface. Consequently, the ions penetrate the cytoplasm, where they interact with the thiol groups of proteins. This phenomenon results in the inactivation of enzymes and disruptions in the functioning of the respiratory chain. The high antimicrobial efficacy of materials containing metal ions is attributed to the combined action of the released ions and ROS [37]. Many authors have sought to link the size of copper oxide nanoparticles with the diameter of the bacterial growth inhibition zone. For example, other authors have reported that for copper concentrations between 125 μg/mL and 1000 μg/mL, the diameter of the inhibition zone for *E. coli* ranged from 13 to 21 mm. For *S. aureus*, these values ranged from 12 to 19 mm [38,39,40]. Similar conclusions were presented earlier by other authors [41,42]. Additionally, it was indicated that copper oxide particles have a better inhibitory effect on Gram-negative bacteria than on Gram-positive bacteria. This may be because Gram-negative bacteria have a negative surface charge and a relatively soft cell wall consisting of a thin layer of peptidoglycan. By contrast, the cell wall of Gram-positive bacteria consists of a thick layer of peptidoglycan, which can hinder the penetration of copper nanoparticles significantly [43]. The results obtained in the present study confirm the antibacterial activity of copper-containing materials. However, the observed effect was predominantly limited to direct contact with the composite surface, suggesting a different mode or extent of antimicrobial action compared with studies reporting pronounced inhibition zones. Other authors have observed larger growth inhibition zones for Gram-negative bacteria compared with Gram-positive bacteria in the presence of silver nanoparticle-modified materials. Numerous studies have shown that growth inhibition zones for *E. coli* are 16–19 mm and for *P. aeruginosa*, 16–20 mm. For Gram-positive *S. aureus* bacteria, the growth inhibition zones were 13–16 mm. The authors also pointed to differences in cell wall structure, noting the presence of a thicker peptidoglycan layer in Gram-negative bacteria, which improves the strength and resistance of bacterial cells [44,45]. A comparable tendency was observed for the silver-modified composites investigated in this study, which generally exhibited stronger antibacterial activity against Gram-negative bacteria than against Gram-positive bacteria. This observation is consistent with previous reports attributing such differences to variations in bacterial cell wall structure and permeability. The mechanism of action of silver nanoparticles is multifaceted. The release of Ag^+^ ions, the induction of oxidative stress, and damage to the cell membrane are among the factors that contribute to this process. Silver ions have been observed to bind to the thiol groups of membrane proteins and enzymes, resulting in a disruption of membrane integrity. Additionally, the process of cellular respiration is hindered, and the balance of ions within the cell is compromised. Following the penetration of the cell by ions, an interaction with DNA ensues, culminating in impaired DNA replication and cell division. This process may be further exacerbated by the generation of ROS [46]. Among all tested formulations, composites containing benzethonium chloride exhibited the most pronounced antibacterial effect. Benzethonium chloride, a quaternary ammonium salt, exhibits a distinct mode of action compared to metal-based antimicrobial agents. The positive ammonium group has been observed to adsorb onto the negatively charged surface of microbial cells and is subsequently incorporated into the lipid bilayer. This results in the disruption of the cytoplasmic membrane by increasing its permeability, which may lead to the leakage of cellular components. Consequently, there is a rapid loss of cell viability, which may simultaneously limit the ability of microorganisms to adhere and form biofilms [27,47]. Their activity was generally greater than that of the materials modified with copper sulfate or silver, regardless of the bacterial strain tested. Interestingly, these materials presented particularly strong activity against *S. aureus*, which contrasts with the trends commonly reported for copper- and silver-based antimicrobial materials, where Gram-negative bacteria are often more susceptible. This suggests that the mechanism responsible for the antibacterial activity of benzethonium chloride-containing composites may differ from those described for metallic antimicrobial agents.

The antifungal activity of composites modified with zinc oxide and copper oxide has been demonstrated by other authors in previous studies [48,49,50]. Zinc oxide is also characterized by a multi-faceted mechanism of action, which involves the generation of reactive oxygen species, the release of Zn^2+^ ions, and the direct interaction of particles with the cell surface. A body of research employing electron paramagnetic resonance (EPR) spectroscopy has evidenced the occurrence of radicals on the surface of zinc oxide (ZnO). This finding serves to substantiate the role of oxidative stress in the antimicrobial activity of materials fortified with zinc oxide. The Zn^2+^ ions that are released may also disrupt the function of enzymes and increase cell membrane permeability, which may result in a potentiation of the antimicrobial effect [51,52]. Growth inhibition zones of 24 mm for *C. albicans* and 21 mm for *A. niger* were determined. Trimetallic composites based on copper, silver, and zinc ions exhibited fairly good antimicrobial activity against *Candida* strains. Growth inhibition zones of approximately 14 mm were determined around the contact surface with the material [53]. Lashin et al. and Javed et al., in their studies, indicate the antibacterial activity of composites containing silica, magnesium oxide, and cerium dioxide. The size, shape, and morphology of the core and coating of the nanostructure have been shown to have the greatest impact on the proliferation of organisms. A high surface-to-volume ratio is important [54,55]. The results obtained in the present study also confirmed the antifungal activity of the investigated composites. However, in contrast to the studies cited above, no visible growth inhibition zones were observed around the tested materials, regardless of the active diluent or antimicrobial modifier used. Instead, fungal growth inhibition was primarily observed directly beneath the composite surface, indicating that the antifungal effect was predominantly contact-dependent.

The antimicrobial activity observed in the present study is likely associated with both direct contact between microorganisms and the composite surface and the gradual release of antimicrobial species from the polymer matrix. In our previous work, the release behavior of nanosilver, copper(II) sulphate, zinc oxide, and benzethonium chloride from analogous methacrylate composites was systematically investigated. The obtained results demonstrated that the release of antimicrobial agents strongly depended on the composition of the polymer matrix and the type of reactive diluent, indicating that the resin formulation plays an important role in controlling the availability of active species. These findings suggest that the differences in antimicrobial activity observed in the present study may be partially attributed to differences in modifier release kinetics. Consequently, optimization of the balance between immobilization and controlled release of antimicrobial agents appears to be essential for achieving sustained antimicrobial efficacy while maintaining the long-term stability and safety of the composites [24].

Studies conducted by other authors in recent years have shown that hydrogel matrices containing copper ions exhibited almost 100% inhibition of the growth of *S. aureus* and *E. coli* bacteria and *C. albicans* fungi. Antimicrobial activity was observed for 72 h, after which it decreased over time. Hydrogel materials modified with zinc ions were found to affect *E. coli*. In this case, a decrease in antimicrobial activity was also observed over time. This has been shown to be related to the process of ion release from the hydrogel matrices [56,57,58]. The results obtained in the present study are in agreement with these observations, confirming that materials containing copper ions exhibit antimicrobial activity against bacterial and fungal strains. Composites with zinc oxide exhibit antimicrobial activity against bacterial strains. However, the effectiveness of the composites depended on the type of active diluent and antimicrobial modifier used. Studies presented by Lange et al., Breijyeh et al., and Slavin et al. have shown that adding nanosilver to materials in amounts up to 25 µg/mL reduces the viability of *E. coli* and *E. faecalis*, and at concentrations above 25 µg/mL reduces the viability of *P. aeruginosa*, *S. aureus*, and *C. albicans*. Modifying materials with copper ions reduced the viability of all microorganisms, whereas ZnO reduced the viability of *E. coli*, *E. faecalis*, and *S. aureus* cells by less than 50% [59,60,61]. A similar broad-spectrum antimicrobial effect was observed for several of the silver- and copper-containing composites investigated in this study. The strongest growth inhibition was generally observed after longer incubation periods, particularly after 24 h of exposure, indicating that the antimicrobial effect of the developed materials increased with contact time.

Siddiqi et al. and Ptasiewicz et al. in their studies indicate that using materials enriched with silver nanoparticles significantly reduces the viability of many pathogens, regardless of their resistance profile. Even at a low concentration of 0.1 mg/mL, silver nanoparticles reduce the number of living cells and inhibit the growth of microorganisms such as *S. aureus*, *P. aeruginosa*, *Cryptococcus*, and *Candida* fungi [62,63]. Copper nanoparticles demonstrated potent growth inhibition and reduced the viability of bacteria and fungi in vitro. Concerning *C. albicans*, this is achieved by increasing oxidative stress and reducing metabolic activity, among other things [64]. Silver, copper, and zinc complexes exhibited significant inhibitory activity against bacteria. This is explained by the interaction of metal ions with microbial cells [65]. Benzethonium chloride exhibits a broad microbicidal spectrum against numerous bacterial and fungal strains due to its ability to disrupt membranes and denature proteins. Zinc oxide nanoparticles exhibited significant antifungal activity against *C. albicans* and *A. niger*, among others. Nanoparticles damage the cell walls and membranes of fungi [66]. Other studies have shown that the mechanism by which nanoparticles act is based on the formation of reactive oxygen species and the consequent loss of fungal membrane integrity [67]. The results obtained in the present study are consistent with these reports and confirm the antimicrobial activity of composites modified with silver, copper, zinc oxide and benzethonium chloride. Most formulations exhibited strong antibacterial activity against *P. aeruginosa* and *E. coli*, whereas a bacteriostatic effect was more frequently observed for *S. aureus*. Furthermore, the high fungicidal activity observed against *C. albicans* and the substantial inhibition of *A. niger* growth support previous findings regarding the antifungal properties of metal-containing materials and ZnO nanoparticles.

The formation of biofilm by bacteria and fungi is a significant clinical and industrial problem. This leads to microorganisms becoming more resistant to antibiotics and disinfectants. Notably, biofilm increases the production of toxins and tissue-degrading enzymes [68]. In the context of medical and industrial materials, it can cause corrosion of surfaces and clogging of filters. When biofilms appear on biomaterials and implants, they increase the risk of complications and chronic infections. Silver nanoparticles can reduce the viability of bacterial and fungal cells through several mechanisms. It has been shown by numerous studies that the use of silver nanoparticles as a modifier has led to a significant reduction in the number of colony-forming units of bacteria and fungi. Additionally, their ability to adhere and form biofilms is inhibited. The size of silver nanoparticles has also been shown to significantly affect their ability to combat biofilm. Smaller particles have a larger surface area, allowing for increased interaction with the biofilm and potentially greater activity in reducing it [69,70]. The authors emphasized the significant role of copper nanoparticles in reducing the risk of biofilm formation by *S. aureus*, *P. aeruginosa*, and *C. albicans*. They indicated that using copper as a modifier can reduce the risk of bacterial biofilm formation by up to 65% and fungal biofilm formation by around 50% [71]. ZnO nanoparticles significantly reduced *Candida* fungal biofilm. It was suggested that this reduction could be as high as 65%. Using smaller nanoparticles significantly improves the surface-to-volume ratio. This allows for better interaction with and penetration of microbial cells. In this case, the mechanism of action of zinc oxide is reported to be the induction of oxidative stress, the disruption of cell membrane structure, and interaction with the cell wall [72,73]. Results obtained in the present study are consistent with these findings, as biofilm formation on the composite surfaces was generally limited. In most cases, no extensive red formazan deposition was observed, indicating reduced microbial adhesion and biofilm development. The few instances of red coloration detected at the edges of selected samples were most likely associated with surface irregularities created during sample preparation rather than with intensive biofilm formation on the composite surface itself.

To facilitate comparison with previous studies, representative antimicrobial methacrylate- and acrylic-based polymer composites reported in the literature are summarized in Table 9. Most published studies have investigated a single polymer formulation containing one antimicrobial additive. In contrast, the present work compares four methacrylate resin compositions modified with four antimicrobial agents representing different mechanisms of action. By evaluating all materials under identical experimental conditions, it was possible to distinguish the influence of the polymer matrix composition from that of the antimicrobial modifier on the antibacterial, antifungal, antibiofilm, and cytocompatibility properties of the developed composites.

Materials used in typical medical applications, e.g., implants, should meet certain key criteria, among which cytotoxicity is crucial. Failure to meet this criterion results in the immediate exclusion of the material from use, as it is constantly exposed to direct contact with used organisms [79]. The conducted cytotoxicity evaluation revealed that composite materials modified with copper sulphate and nanosilver presented toxic effects towards BJ cells. These data confirm the previously described effects of copper ions and nanosilver. Ag nanoparticles as well as Cu ions are known to possess cytotoxic, genotoxic, and antiproliferative impacts on normal human cells [80,81]. According to the ISO 10993-5, materials are considered toxic if cell viability is reduced below 70% [31]. Based on this recommendation, it can be concluded that the remaining samples used in the work, modified with zinc oxide and benzethonium chloride, were non-toxic. An important finding of the present study is that composites modified with zinc oxide and benzethonium chloride combined high antimicrobial activity with good cytocompatibility toward normal human skin fibroblasts. This observation suggests that these modifiers provide a favorable balance between biological safety and antimicrobial efficacy, which is essential for materials intended for biomedical applications. Zinc oxide is considered a relatively biocompatible antimicrobial agent because zinc is an essential trace element involved in numerous cellular processes, while its antimicrobial activity results primarily from controlled Zn^2+^ ion release and ROS generation [82]. In turn, benzethonium chloride-containing composites exhibited particularly strong antibacterial activity without compromising fibroblast viability. This may indicate that incorporation of benzethonium chloride into the crosslinked polymer matrix limited its direct cytotoxic effects while maintaining sufficient antimicrobial activity at the material surface. Consequently, both modifiers appear to be promising candidates for the development of multifunctional methacrylate composites combining effective antimicrobial protection with acceptable biological safety [83].

## 5. Conclusions

The present study demonstrated that methacrylate-based composites modified with nanosilver, copper sulfate, benzethonium chloride, and zinc oxide exhibit a broad spectrum of antibacterial and antifungal activity, effectively reducing the viability of microorganisms and inhibiting biofilm formation. The findings confirmed that the biological properties of the developed composites were contingent not only on the antimicrobial modifier but also on the type of reactive diluent utilized in the polymer matrix. This underscores the significance of matrix composition in the design of methacrylate materials with antimicrobial properties.

Among the modifiers examined, benzethonium chloride demonstrated the most pronounced antibacterial activity, while composites comprising zinc oxide and benzethonium chloride exhibited the most favorable cytocompatibility with normal human skin fibroblasts. Furthermore, composites modified with silver and copper compounds demonstrated notable antibacterial activity, though the efficacy of these composites varied depending on the microorganism tested and the resin composition.

A discussion of the results obtained, supported by the current state of the literature, indicates that the antibacterial activity of the developed composites is most likely associated with a number of complementary mechanisms, including direct damage to the cell membrane upon contact, the release of metal ions, oxidative stress induced by reactive oxygen species, and the inhibition of microbial metabolic processes and biofilm formation. From an application perspective, it will be important to conduct further research on the conditions of use like evaluating whether the antimicrobial activity is maintained after prolonged storage, repeated washing, or environmental exposure.

In summary, the synthesized methacrylate composites manifest as promising multifunctional materials, exhibiting considerable potential for utilization in biomedical devices, protective coatings, and other technical or industrial domains necessitating long-term management of microbial contamination.

## 6. Patent Applications

Młynarczyk, K.; Podkościelna, B.; Osińska-Jaroszuk, M.; Jaszek, M. Method of Obtaining a Polymeric Composite with Antimicrobial Activity and a Polymeric Composite Obtained by this Method. P. 444108, 15 March 2023.

Młynarczyk, K., Podkościelna, B., Osińska-Jaroszuk, M., and Jaszek, M. Polymer Composites with Antibacterial Properties. P.447892, 28 February 2024.

## Figures and Tables

**Table 1:** Antimicrobial activity of NVP-containing composites evaluated on agar plates against Gram-positive and Gram-negative bacteria. Zones of inhibition are given in cm.

Microorganism/Composite	*P. aeruginosa*	*E. coli*	*S. aureus*
BPA.DM + NVP *	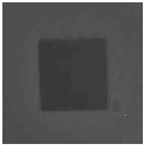	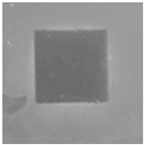	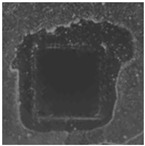
	0 cm	0 cm	0.2 cm
BPA.DM + NVP + BEN	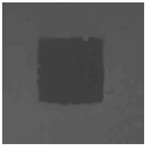	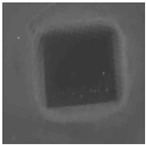	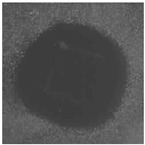
	0.3 cm	0.4 cm	0.6 cm
BPA.DM + NVP + CuSO_4_	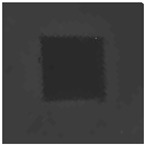	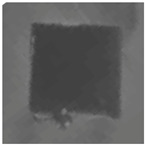	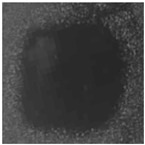
	0.1 cm	0 cm	0.2 cm
BPA.DM + NVP + Ag	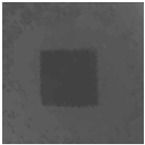	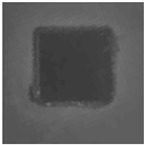	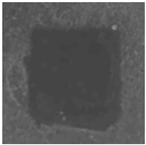
	0.4 cm	0.2 cm	0 cm

* Note: The results for the unmodified composites (control samples) were previously reported in Ref. [25] and are included here to allow direct comparison with composites modified using different antimicrobial agents.

**Table 2:** Antimicrobial activity of NVP-containing composites evaluated on agar plates against fungi. Zones of inhibition are given in cm.

Microorganism/Composite	*C. albicans*	*A. niger*
BPA.DM + NVP	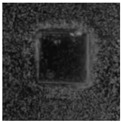 0 cm	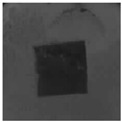 0 cm
BPA.DM + NVP + BEN	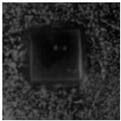 0 cm	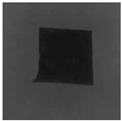 0 cm
BPA.DM + NVP + CuSO_4_	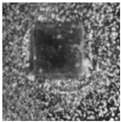 0 cm	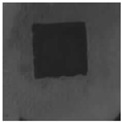 0 cm
BPA.DM + NVP + Ag	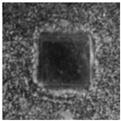 0 cm	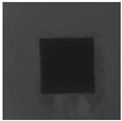 0 cm
BPA.DM + NVP + ZnO	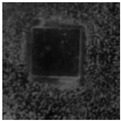 0 cm	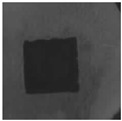 0 cm

**Table 3:** Analysis of bacterial growth inhibition [%] in liquid cultures in the presence of NVP-containing composites using the serial dilution method. Data presented as mean value with SD.

Microorganism /Composite	*P. aeruginosa*		*E. coli*		*S. aureus*	
	After 12 h	After 24 h	After 12 h	After 24 h	After 12 h	After 24 h
BPA.DM + NVP	17.8 ± 1.9	59.4 ± 2.2	40.0 ± 0.7	53.5 ± 2.2	29.4 ± 2.0	47.9 ± 2.2
BPA.DM + NVP + BEN	73.8 ± 0.3	95.3 ± 1.7	57.3 ± 0.1	15.3 ± 0.3	57.4 ± 0.4	50.7 ± 1.0
BPA.DM + NVP + CuSO_4_	30.4 ± 1.0	49.2 ± 0.4	28.8 ± 1.4	7.0 ± 0.2	37.2 ± 0.9	20.3 ± 0.1
BPA.DM + NVP + Ag	67.4 ± 1.2	16.9 ± 0.1	55.1 ± 1.1	12.4 ± 0.4	11.2 ± 0.2	19.5 ± 0.1

Note: Values corresponding to growth inhibition exceeding 45% are highlighted in grey.

**Table 4:** Analysis of fungal growth inhibition [%] in liquid cultures in the presence of NVP-containing composites using the serial dilution method. Data presented as mean value with SD.

Microorganism/ Composite	*C. albicans*		*A. niger*	
	After 12 h	After 24 h	After 12 h	After 24 h
BPA.DM + NVP	73.5 ± 1.8	22.0 ± 0.1	79.8 ± 1.3	86.9 ± 2.1
BPA.DM + NVP + BEN	58.8 ± 0.2	34.0 ± 0.4	72.0 ± 0.4	63.3 ± 0.9
BPA.DM + NVP + CuSO_4_	80.3 ± 0.9	18.8 ± 0.2	77.3 ± 1.3	61.9 ± 1.2
BPA.DM + NVP + Ag	60.6 ± 0.5	76.8 ± 0.8	89.8 ± 0.7	61.8 ± 1.2
BPA.DM + NVP + ZnO	80.1 ± 1.4	65.6 ± 0.6	68.5 ± 0.3	46.1 ± 0.4

Note: Values corresponding to growth inhibition exceeding 45% are highlighted in grey.

**Table 5:** Detection of bacterial survival rate in the presence of NVP-containing composite using the method with TTC as a reaction substrate.

Microorganism/Composite	*P. aeruginosa*	*E. coli*	*S. aureus*
Growth control *	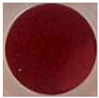	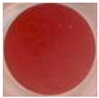	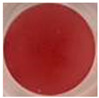
BPA.DM + NVP *	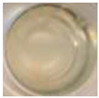	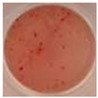	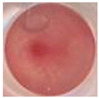
BPA.DM + NVP + BEN	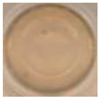	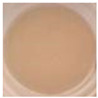	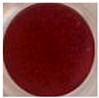
BPA.DM + NVP + CuSO_4_	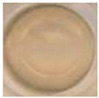	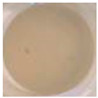	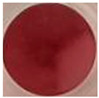
BPA.DM + NVP + Ag	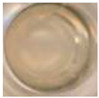	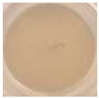	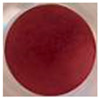

* Note: The unmodified composites and the growth control correspond to the control data previously published in Ref. [25]. They are presented here to provide a common reference for comparison with composites modified using different antimicrobial agents.

**Table 6:** Detection of fungal survival rate in the presence of NVP-containing composite using the method with TTC as a reaction substrate.

Microorganism/Composite	*C. albicans*	*A. niger*
Growth control	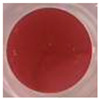	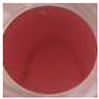
BPA.DM + NVP	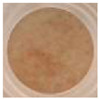	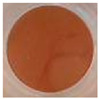
BPA.DM + NVP + BEN	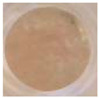	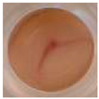
BPA.DM + NVP + CuSO_4_	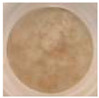	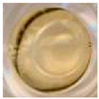
BPA.DM + NVP + Ag	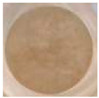	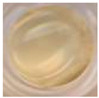
BPA.DM + NVP + ZnO	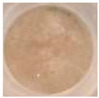	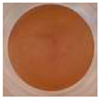

**Table 7:** Evaluation of the bacterial biofilm formation on the surface of NVP-containing composites using TTC as a reaction substrate.

Microorganism/Composite	*P. aeruginosa*	*E. coli*	*S. aureus*
BPA.DM + NVP *	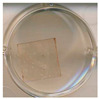	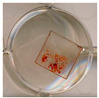	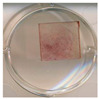
BPA.DM + NVP + BEN	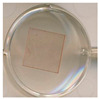	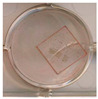	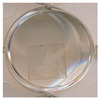
BPA.DM + NVP + CuSO_4_	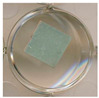	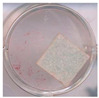	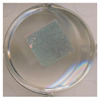
BPA.DM + NVP + Ag	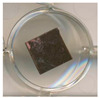	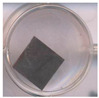	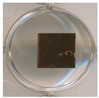

* Note: The results for the unmodified composites (control samples) were previously reported in Ref. [25] and are included here to allow direct comparison with composites modified using different antimicrobial agents.

**Table 8:** Evaluation of the fungal biofilm formation on the surface of NVP-containing composites using TTC as a reaction substrate.

Microorganism/Composite	*C. albicans*	*A. niger*
BPA.DM + NVP	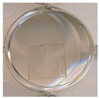	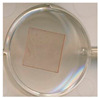
BPA.DM + NVP + BEN	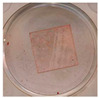	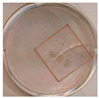
BPA.DM + NVP + CuSO_4_	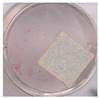	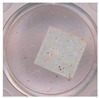
BPA.DM + NVP + Ag	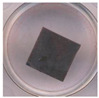	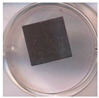
BPA.DM + NVP + ZnO	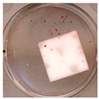	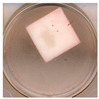

**Table 9:** Comparison of biological evaluation performed in representative antimicrobial methacrylate-based composites.

Polymer Matrix	Modifier	Tested Microorganisms	Antimicrobial Performance
PMMA	Ag/ZnO	*E. coli*	Strong antimicrobial activity [74]
Cross-linked methacrylate resin	Ag	*S. aureus*, *E. coli*	Significant antibacterial activity while maintaining mechanical properties [75]
Cross-linked methacrylate resin	Quaternary ammonium methacrylate	*S. mutans*, *E. coli*	Strong contact-active antibacterial activity with negligible leaching [76]
Cross-linked PMMA	CuO	*E. coli*, *S. aureus*	CuO improved antibacterial activity, particularly against *S. aureus* [77]
PMMA	ZnO, CuO	*S. aureus*, *C. albicans*	ZnO showed pronounced antibacterial and antifungal activity, whereas CuO exhibited considerably lower antimicrobial efficacy [78]
Our study: BPA.DM + HEMA/NVP/MMA/AEH	Ag, CuSO_4_, ZnO, benzethonium chloride	*S. aureus*, *E. coli*, *P. aeruginosa*, *C. albicans*, *A. niger*	BEN-containing composites exhibited the strongest antibacterial activity (particularly against *P. aeruginosa* and *S. aureus*). Selected ZnO- and CuSO_4_-containing composites showed >80% inhibition of *C. albicans*, whereas Ag- and CuSO_4_-modified materials achieved >90% inhibition of *A. niger*. ZnO- and BEN-containing composites demonstrated the most favorable balance between antimicrobial activity and cytocompatibility.

## Data Availability

The data presented in this study are available on request from the corresponding authors. The data are not publicly available due to privacy concerns.

## Supplementary Materials

The following supporting information can be downloaded at https://www.mdpi.com/article/10.3390/ma19143053/s1, Table S1. Antimicrobial activity of AEH-containing composites evaluated on agar plates against Gram-positive and Gram-negative bacteria. Zones of inhibition are given in cm. Table S2. Antimicrobial activity of HEMA-containing composites evaluated on agar plates against Gram-positive and Gram-negative bacteria. Zones of inhibition are given in cm. Table S3. Antimicrobial activity of MMA-containing composites evaluated on agar plates against Gram-positive and Gram-negative bacteria. Zones of inhibition are given in cm. Table S4. Antimicrobial activity of AEH-containing composites evaluated on agar plates against fungi. Zones of inhibition are given in cm. Table S5. Antimicrobial activity of HEMA-containing composites evaluated on agar plates against fungi. Zones of inhibition are given in cm. Table S6. Antimicrobial activity of MMA-containing composites evaluated on agar plates against fungi. Zones of inhibition are given in cm. Table S7. Analysis of bacterial growth inhibition [%] in liquid cultures in the presence of AEH-, HEMA-, and MMA-containing composites using the serial dilution method. Data presented as mean value with SD. Values higher than 45% are marked in grey. Table S8. Analysis of fungal growth inhibition [%] in liquid cultures in the presence of AEH-, HEMA-, and NVP-containing composites using the serial dilution method. Data presented as mean value with SD. Values higher than 45% are marked in grey. Table S9. Detection of bacterial survival rate in the presence of AEH-, HEMA-, and MMA-containing composite using the method with TTC as a reaction substrate. Table S10. Detection of fungal survival rate in the presence of AEH-, HEMA-, and MMA-containing composite using the method with TTC as a reaction substrate. Table S11. Evaluation of the bacterial biofilm formation on the surface of AEH-, HEMA-, and MMA-containing composites using TTC as reaction substrate. Table S12. Evaluation of the fungal biofilm formation on the surface of AEH-, HEMA-, and MMA-containing composites using TTC as reaction substrate.

## Abbreviations

The following abbreviations are used in this manuscript:

BPA.DM	Bisphenol A glycerolate dimethacrylate
AEH	2-ethylhexyl acrylate
MMA	Methyl methacrylate
NVP	1-vinyl-2-pyrrolidone
HEMA	2-hydroxyethyl methacrylate
BEN	Benzethonium chloride
Ag	Nanosilver
CuSO_4_	Copper(II) sulphate
ZnO	Zinc oxide
IQ	Irgacure 651
TTC	2,3,5-triphenyltetrazolium chloride
BPA.DM + AEH	Composite material containing 2-ethylhexyl acrylate
BPA.DM + AEH + BEN	Composite material containing 2-ethylhexyl acrylate and benzethonium chloride
BPA.DM + AEH + Ag	Composite material containing 2-ethylhexyl acrylate and nanosilver
BPA.DM + AEH + CuSO_4_	Composite material containing 2-ethylhexyl acrylate and copper(II) sulphate
BPA.DM + AEH + ZnO	Composite material containing 2-ethylhexyl acrylate and zinc oxide
BPA.DM + MMA	Composite material containing methyl methacrylate
BPA.DM + MMA + BEN	Composite material containing methyl methacrylate and benzethonium chloride
BPA.DM + MMA + Ag	Composite material containing methyl methacrylate and nanosilver
BPA.DM + MMA+ CuSO_4_	Composite material containing methyl methacrylate and copper(II) sulphate
BPA.DM + MMA + ZnO	Composite material containing methyl methacrylate and zinc oxide
BPA.DM + NVP	Composite material containing 1-vinyl-2-pyrrolidone
BPA.DM + NVP + BEN	Composite material containing 1-vinyl-2-pyrrolidone and benzethonium chloride
BPA.DM + NVP + Ag	Composite material containing 1-vinyl-2-pyrrolidone and nanosilver
BPA.DM + NVP + CuSO_4_	Composite material containing 1-vinyl-2-pyrrolidone and copper(II) sulphate
BPA.DM + NVP + ZnO	Composite material containing 1-vinyl-2-pyrrolidone and zinc oxide
BPA.DM + HEMA	Composite material containing 2-hydroxyethyl methacrylate
BPA.DM + HEMA + BEN	Composite material containing 2-hydroxyethyl methacrylate and benzethonium chloride
BPA.DM + HEMA + Ag	Composite material containing 2-hydroxyethyl methacrylate and nanosilver
BPA.DM + HEMA+ CuSO_4_	Composite material containing 2-hydroxyethyl methacrylate and copper(II) sulphate
BPA.DM + HEMA + ZnO	Composite material containing 2-hydroxyethyl methacrylate and zinc oxide
