# Supplementary material for: Novel Antimicrobial Composites Modified with Nanosilver, CuSO4, Benzethonium Chloride, and ZnO
Source: Materials (Basel). 2026 Jul 15;19(14):3053. doi: 10.3390/ma19143053 (PMC13413231; doi:10.3390/ma19143053)
Supplement: Supplementary file 1 [file materials-19-03053-s001.zip › materials-4400992-supplementary.pdf]

materials

Article

Novel Antimicrobial Composites Modified with Nanosilver, $CuSO_4$, Benzethonium Chloride, and ZnO

Karolina Kiełczewska-Klim [1,*], Beata Podkościelna [1,*], Katarzyna Szałapata [2], Monika Osińska-Jaroszuk [2], Vladyslav Vivcharenko [3], and Magdalena Jaszek [2]

[1] Department of Polymer Chemistry, Maria Curie-Sklodowska University in Lublin, Gliniana 33, 20-614 Lublin, Poland
[2] Department of Biochemistry and Biotechnology, Maria Curie-Sklodowska University in Lublin, Akademicka 19, 20-033 Lublin, Poland; katarzyna.szalapata@mail.umcs.pl (K.S.); monika.osinska-jaroszuk@mail.umcs.pl (M.O.-J.); magdalena.jaszek@mail.umcs.pl (M.J.)
[3] Department of Tissue Engineering and Regenerative Medicine, Medical University of Lublin, Chodzki 1, 20-093 Lublin, Poland; vladyslav.vivcharenko@umlub.edu.pl
* Correspondence: karolina.mlynarczyk@mail.umcs.pl (K.K.-K.); beata.podkoscielna@mail.umcs.pl (B.P.)

Keywords: antimicrobial properties; biofilm formation; cytotoxicity; bactericidal and fungicidal activity; modified cross-linked methacrylate polymer materials; special additives

Table S1. Antimicrobial activity of AEH-containing composites evaluated on agar plates against Gram-positive and Gram-negative bacteria. Zones of inhibition are given in cm.

**Microorganism / composite**	***P. aeruginosa***	***E. coli***	***S. aureus***
BPA.DM+AEH*	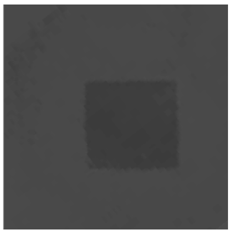<br>0 cm	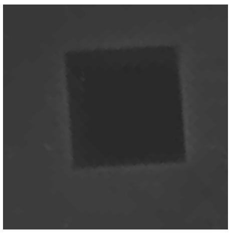<br>0 cm	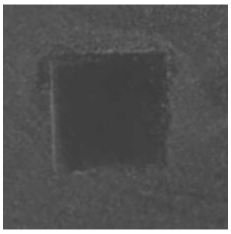<br>0 cm
BPA.DM+AEH+BEN	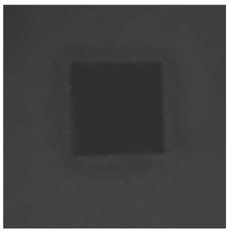<br>0.3 cm	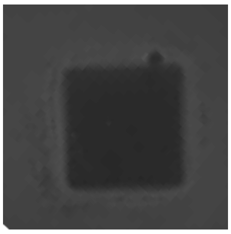<br>0.3 cm	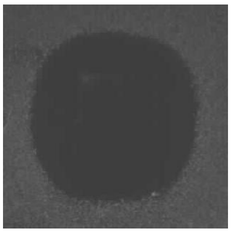<br>0.6 cm
BPA.DM+AEH+$CuSO_4$	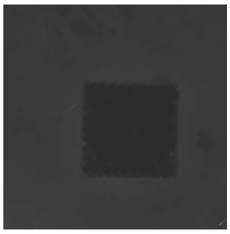<br>0.1 cm	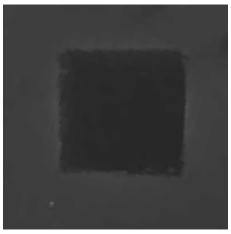<br>0.1 cm	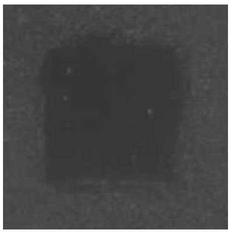<br>0.1 cm
BPA.DM+AEH+Ag	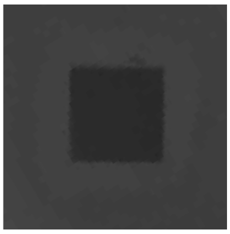<br>0.3 cm	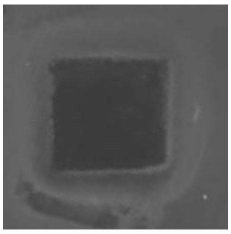<br>0.4 cm	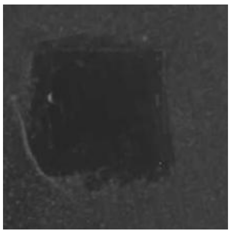<br>0 cm

*Note: The results for the unmodified composites (control samples) were previously reported in Ref. [25] and are included here to allow direct comparison with composites modified using different antimicrobial agents.

https://doi.org/10.3390/ma19143053

Table S2. Antimicrobial activity of HEMA-containing composites evaluated on agar plates against Gram-positive and Gram-negative bacteria. Zones of inhibition are given in cm.

**Microorganism / composite**	***P. aeruginosa***	***E. coli***	***S. aureus***
BPA.DM+HEMA*	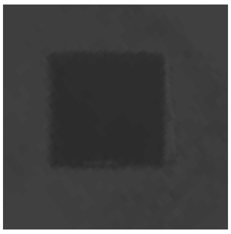	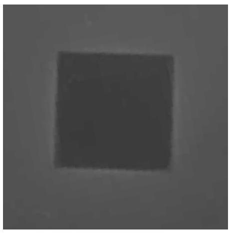	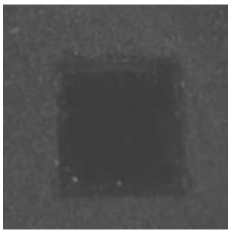
	0 cm	0 cm	0 cm
BPA.DM+HEMA+BEN	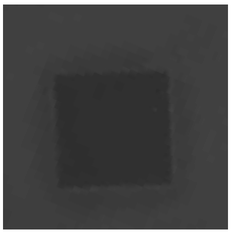	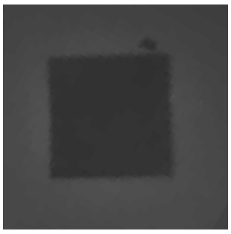	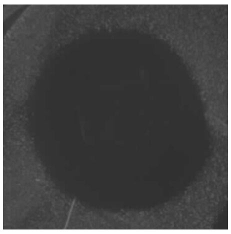
	0.3 cm	0.2 cm	0.7 cm
BPA.DM+HEMA+$CuSO_4$	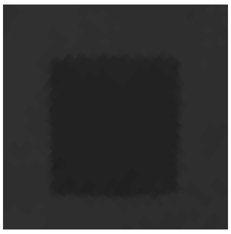	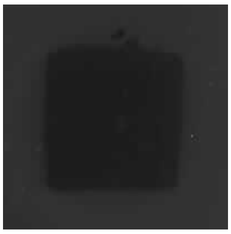	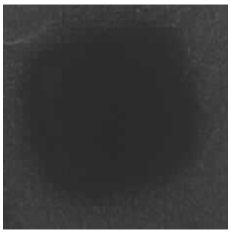
	0 cm	0 cm	0.1 cm
BPA.DM+HEMA+Ag	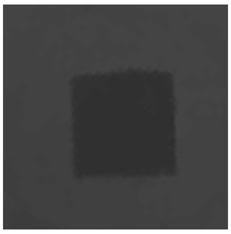	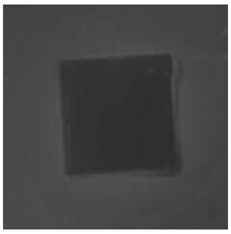	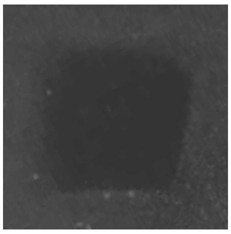
	0.3 cm	0.1 cm	0 cm

*Note: The results for the unmodified composites (control samples) were previously reported in Ref. [25] and are included here to allow direct comparison with composites modified using different antimicrobial agents.

Table S3. Antimicrobial activity of MMA-containing composites evaluated on agar plates against Gram-positive and Gram-negative bacteria. Zones of inhibition are given in cm.

**Microorganism / composite**	***P. aeruginosa***	***E. coli***	***S. aureus***
BPA.DM+MMA*	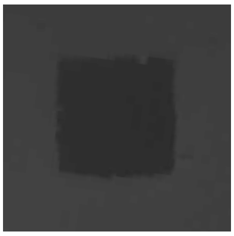	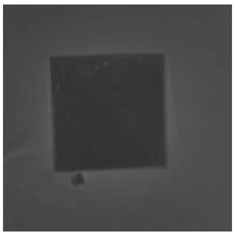	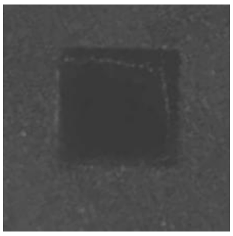
	0 cm	0 cm	0 cm
BPA.DM+MMA+BEN	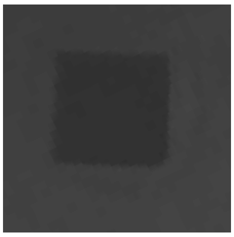	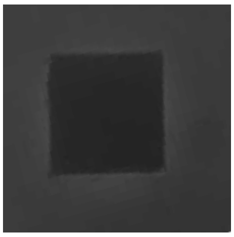	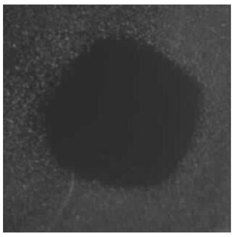
	0.4 cm	0.3 cm	0.3 cm
BPA.DM+MMA+$CuSO_4$	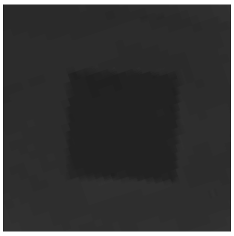	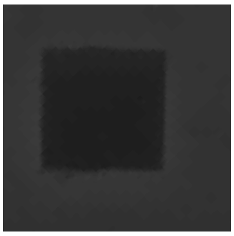	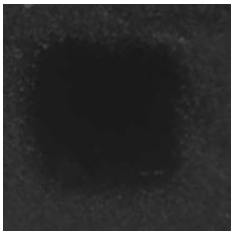
	0.1 cm	0.1 cm	0.1 cm
BPA.DM+MMA+Ag	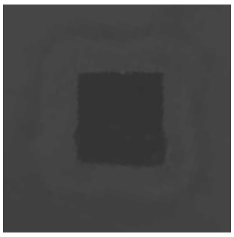	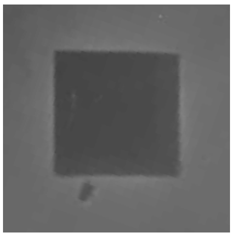	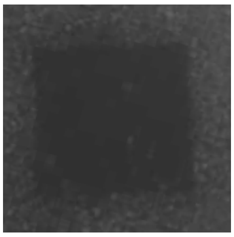
	0.3 cm	0.1 cm	0 cm

*Note: The results for the unmodified composites (control samples) were previously reported in Ref. [25] and are included here to allow direct comparison with composites modified using different antimicrobial agents.

Table S4. Antimicrobial activity of AEH-containing composites evaluated on agar plates against fungi. Zones of inhibition are given in cm.

**Microorganism / composite**	***C. albicans***	***A. niger***
BPA.DM+AEH	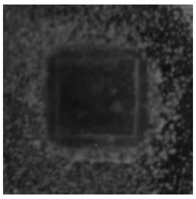 0 cm	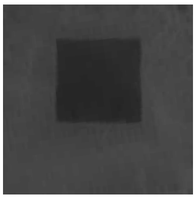 0 cm
BPA.DM+AEH+BEN	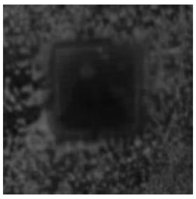 0 cm	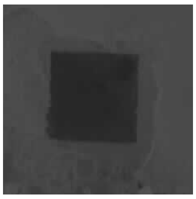 0 cm
BPA.DM+AEH+$CuSO_4$	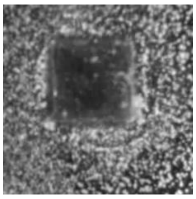 0 cm	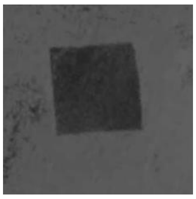 0 cm
BPA.DM+AEH+Ag	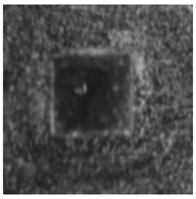 0 cm	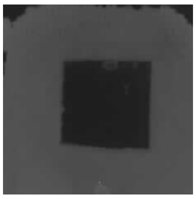 0 cm
BPA.DM+AEH+ZnO	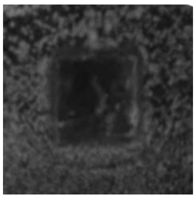 0 cm	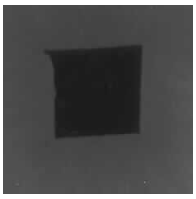 0 cm

Table S5. Antimicrobial activity of HEMA-containing composites evaluated on agar plates against fungi. Zones of inhibition are given in cm.

**Microorganism / composite**	***C. albicans***	***A. niger***
BPA.DM+HEMA	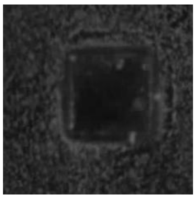<br>0 cm	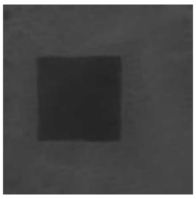<br>0 cm
BPA.DM+HEMA+BEN	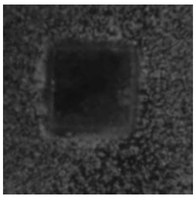<br>0 cm	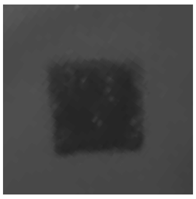<br>0 cm
BPA.DM+HEMA+$CuSO_4$	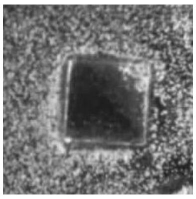<br>0 cm	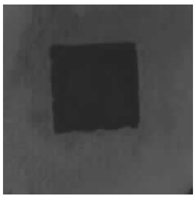<br>0 cm
BPA.DM+HEMA+Ag	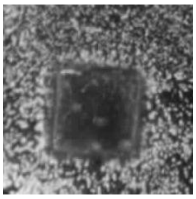<br>0 cm	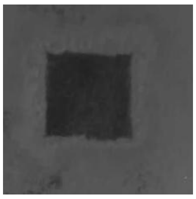<br>0 cm
BPA.DM+HEMA+ZnO	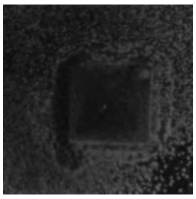<br>0 cm	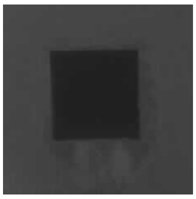<br>0 cm

https://doi.org/10.3390/ma19143053

Table S6. Antimicrobial activity of MMA-containing composites evaluated on agar plates against fungi. Zones of inhibition are given in cm.

Microorganism / composite	*C. albicans*	*A. niger*
BPA.DM+MMA	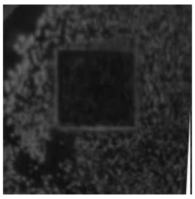<br>0 cm	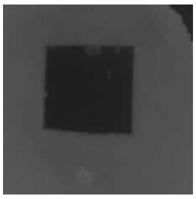<br>0 cm
BPA.DM+MMA+BEN	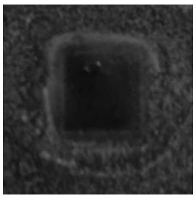<br>0 cm	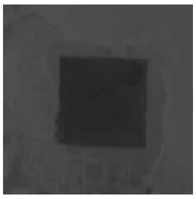<br>0 cm
BPA.DM+MMA+$CuSO_4$	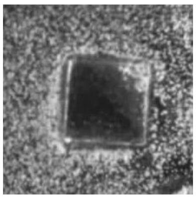<br>0 cm	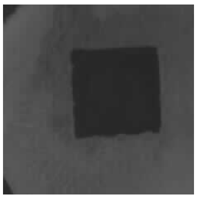<br>0 cm
BPA.DM+MMA+Ag	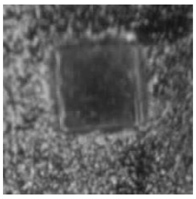<br>0 cm	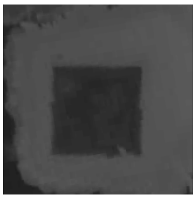<br>0 cm
BPA.DM+MMA+ZnO	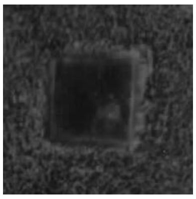<br>0 cm	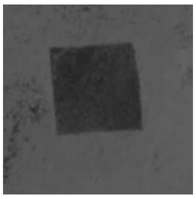<br>0 cm

https://doi.org/10.3390/ma19143053

Table S7. Analysis of bacterial growth inhibition [%] in liquid cultures in the presence of AEH-, HEMA-, and MMA-containing composites using the serial dilution method. Data presented as mean value with SD.

Microorganism /composite	*P. aeruginosa*		*E. coli*		*S. aureus*	
	After 12 hours	After 24 hours	After 12 hours	After 24 hours	After 12 hours	After 24 hours
BPA.DM+AEH	39.6±1.6	40.5±2.0	47.0±3.1	48.0±0.9	32.4±1.4	43.6±1.3
BPA.DM+AEH +BEN	52.2±1.7	78.9±2.1	42.4±0.9	20.5±1.1	65.8±0.7	78.5±0.3
BPA.DM+AEH +$CuSO_4$	24.6±0.6	55.7±2.7	28.3±1.1	2.1±0.1	12.2±0.1	7.5±0.2
BPA.DM+AEH +Ag	54.2±0.3	73.9±0.7	31.5±0.6	25.8±0.1	38.6±0.7	39.3±1.1
BPA.DM+HEMA	22.5±1.4	40.7±1.8	34.6±0.6	36.9±0.4	18.3±0.6	48.6±1.2
BPA.DM+HEMA +BEN	50.6±0.4	75.8±2.9	46.1±1.4	17.8±0.7	70.1±0.3	85.5±2.1
BPA.DM+HEMA +$CuSO_4$	22.7±0.4	66.3±2.1	18.2±0.1	16.6±0.4	16.0±0.1	21.5±0.3
BPA.DM+HEMA +Ag	33.6±0.4	49.5±0.7	37.1±1.0	42.9±2.5	15.6±0.4	51.7±2.1
BPA.DM+MMA	5.5±0.9	73.4±0.9	39.5±0.4	33.4±1.2	7.5±0.2	56.3±0.4
BPA.DM+MMA +BEN	23.6±0.1	88.8±2.3	63.1±0.9	17.8±0.8	52.7±1.4	67.3±2.0
BPA.DM+MMA +$CuSO_4$	6.6±0.4	38.1±1.2	22.7±0.2	16.6±1.1	12.1±0.9	8.5±0.1
BPA.DM+MMA +Ag	54.5±1.1	70.3±1.2	44.6±0.2	20.8±0.1	20.1±0.3	74.6±0.4

Note: Values corresponding to growth inhibition exceeding 45% are highlighted in grey.

Table S8. Analysis of fungal growth inhibition [%] in liquid cultures in the presence of AEH-, HEMA-, and NVP-containing composites using the serial dilution method. Data presented as mean value with SD.

Microorganism / composite	*C. albicans*		*A. niger*	
	After 12 hours	After 24 hours	After 12 hours	After 24 hours
BPA.DM+AEH	72.7±1.4	23.7±0.4	83.5±1.4	80.6±0.6
BPA.DM+AEH +BEN	4.1±0.1	36.9±1.2	84.5±0.1	79.3±0.4
BPA.DM+AEH +$CuSO_4$	66.3±2.3	10.9±0.1	84.4±1.2	91.9±1.9
BPA.DM+AEH +Ag	51.2±1.1	55.4±0.4	89.4±0.3	76.6±0.5
BPA.DM+AEH +ZnO	77.2±2.0	56.8±0.6	75.7±0.5	81.7±0.1
BPA.DM+HEMA	66.6±0.6	4.9±0.2	89.2±0.6	85.5±0.3
BPA.DM+HEMA +BEN	60.5±0.4	38.7±0.7	89.4±0.1	86.2±1.7
BPA.DM+HEMA +$CuSO_4$	81.4±0.3	6.7±0.3	91.1±1.7	84.7±0.2
BPA.DM+HEMA +Ag	40.8±0.9	61.1±0.1	95.4±0.3	87.5±0.7
BPA.DM+HEMA +ZnO	56.2±1.2	38.3±1.2	89.2±1.9	56.6±0.6
BPA.DM+MMA	69.8±0.4	22.8±0.6	84.8±0.4	82.2±1.2
BPA.DM+MMA +BEN	65.2±1.8	40.9±0.4	86.5±0.2	83.4±1.4
BPA.DM+MMA +$CuSO_4$	74.8±0.7	19.7±0.7	88.4±1.6	78.3±0.6
BPA.DM+MMA +Ag	40.0±0.1	54.2±0.3	95.6±1.7	86.1±0.4
BPA.DM+MMA +ZnO	67.7±0.3	6.1±0.1	85.1±0.4	82.9±1.2

Note: Values corresponding to growth inhibition exceeding 45% are highlighted in grey.

Table S9. Detection of bacterial survival rate in the presence of AEH-, HEMA-, and MMA--containing composite using the method with TTC as a reaction substrate

**Microorganism / composite**	***P. aeruginosa***	***E. coli***	***S. aureus***
Growth control*	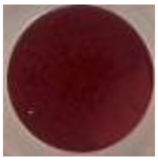	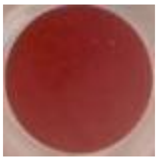	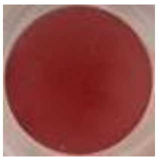
BPA.DM<br>+AEH*	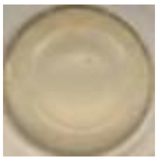	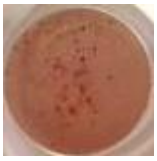	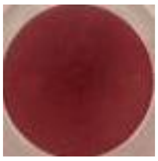
BPA.DM+AEH<br>+BEN	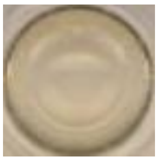	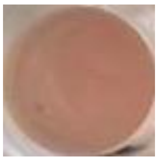	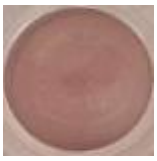
BPA.DM+AEH<br>+$CuSO_4$	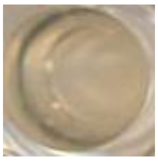	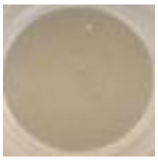	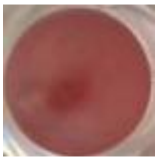
BPA.DM+AEH<br>+Ag	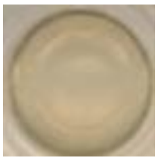	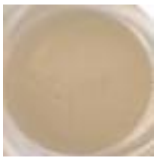	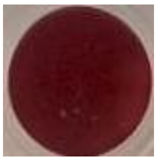
BPA.DM+HEMA*	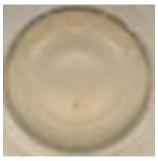	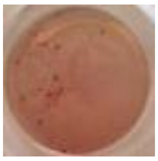	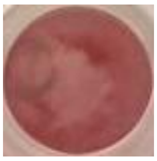
BPA.DM+HEMA<br>+BEN	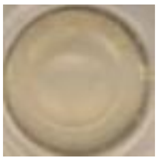	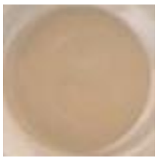	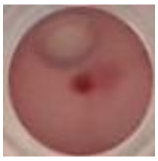
BPA.DM+HEMA<br>+$CuSO_4$	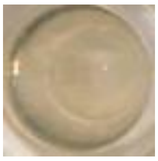	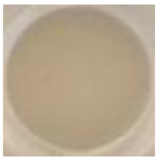	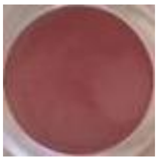
BPA.DM+HEMA<br>+Ag	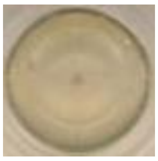	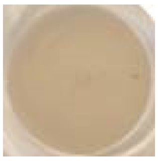	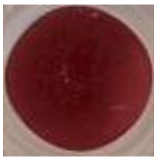
BPA.DM+MMA*	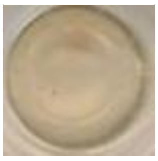	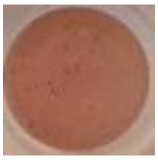	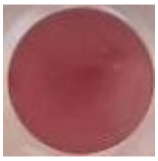

https://doi.org/10.3390/ma19143053

BPA.DM+MMA +BEN	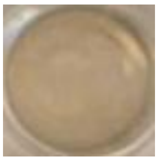	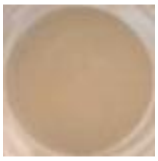	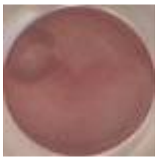
BPA.DM+MMA +$CuSO_4$	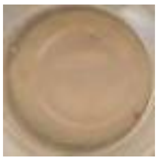	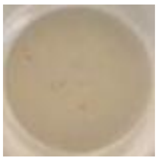	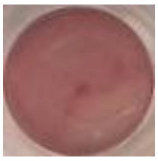
BPA.DM+MMA +Ag	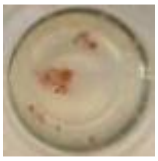	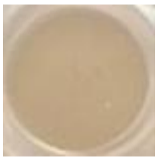	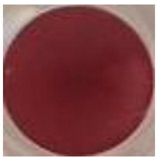

Note: The unmodified composites and the growth control correspond to the control data previously published in Ref. 25. They are presented here to provide a common reference for comparison with composites modified using different antimicrobial agents.

https://doi.org/10.3390/ma19143053

Table S10. Detection of fungal survival rate in the presence of AEH-, HEMA-, and MMA-containing composite using the method with TTC as a reaction substrate

Microorganism / composite	*C. albicans*	*A. niger*
Growth control	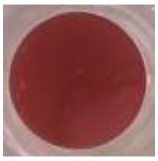	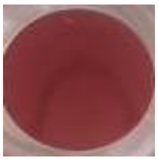
BPA.DM+AEH	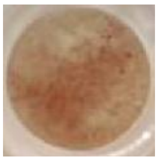	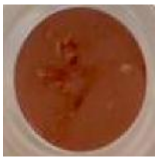
BPA.DM+AEH +BEN	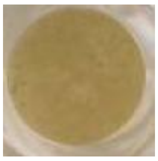	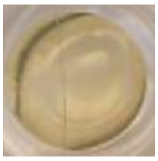
BPA.DM+AEH +$CuSO_4$	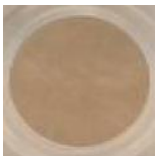	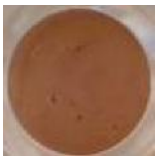
BPA.DM+AEH +Ag	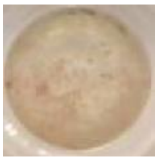	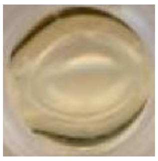
BPA.DM+AEH +ZnO	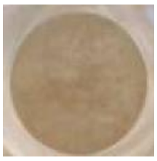	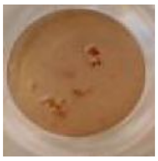
BPA.DM+HEMA	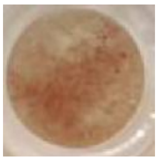	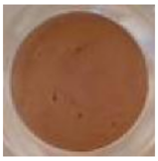
BPA.DM+HEMA +BEN	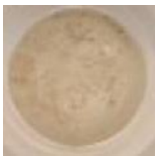	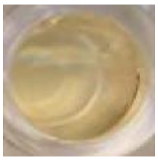
BPA.DM+HEMA +$CuSO_4$	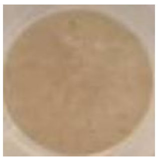	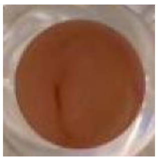
BPA.DM+HEMA +Ag	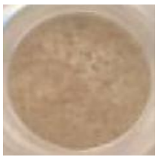	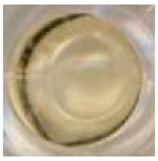

https://doi.org/10.3390/ma19143053

BPA.DM+HEMA +ZnO	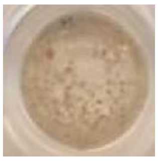	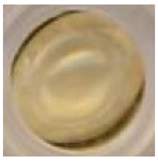
BPA.DM+MMA	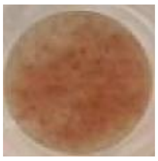	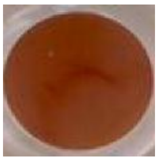
BPA.DM+MMA +BEN	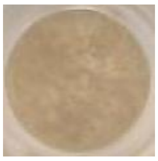	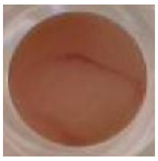
BPA.DM+MMA +$CuSO_4$	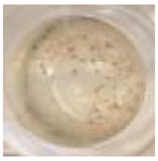	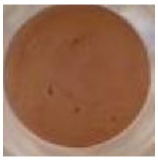
BPA.DM+MMA +Ag	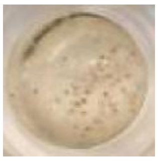	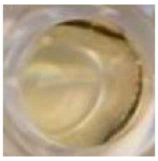
BPA.DM+MMA +ZnO	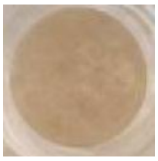	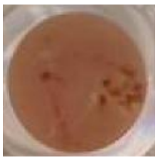

Table S11. Evaluation of the bacterial biofilm formation on the surface of AEH-, HEMA-, and MMA-containing composites using TTC as reaction substrate

Microorganism / composite	*P. aeruginosa*	*E. coli*	*S. aureus*
BPA.DM<br>+AEH*	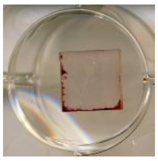	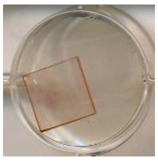	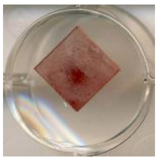
BPA.DM<br>+AEH<br>+BEN	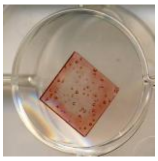	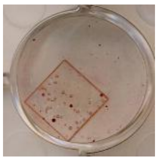	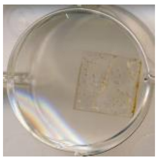
BPA.DM<br>+AEH<br>+$CuSO_4$	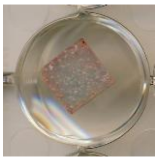	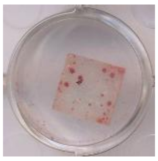	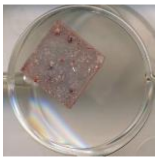
BPA.DM<br>+AEH<br>+Ag	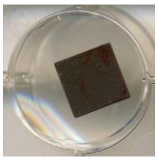	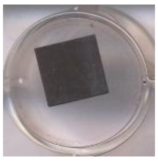	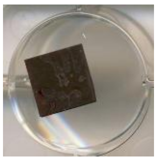
BPA.DM<br>+HEMA*	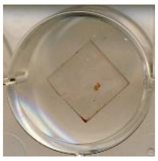	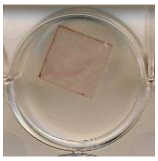	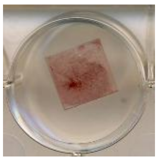
BPA.DM<br>+HEMA<br>+BEN	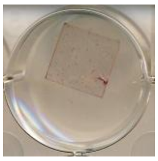	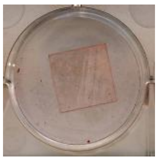	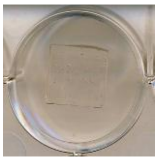
BPA.DM<br>+HEMA<br>+$CuSO_4$	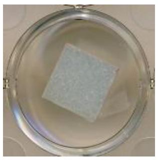	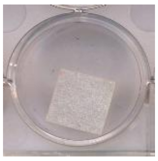	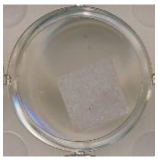
BPA.DM<br>+HEMA<br>+Ag	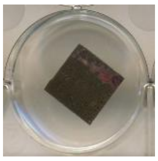	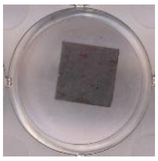	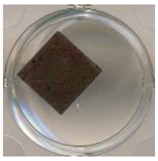
BPA.DM<br>+MMA*	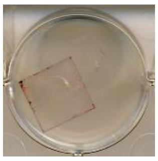	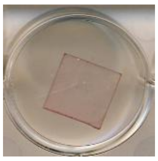	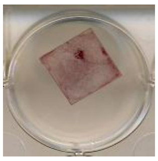
BPA.DM<br>+MMA<br>+BEN	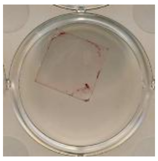	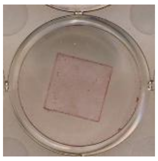	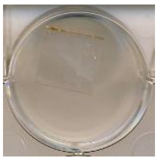

https://doi.org/10.3390/ma19143053

BPA.DM<br>+MMA<br>+$CuSO_4$	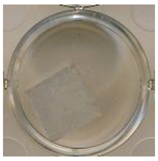	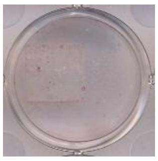	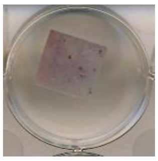
BPA.DM<br>+MMA<br>+Ag	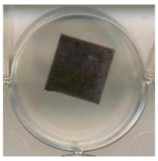	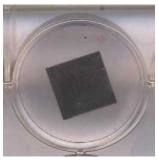	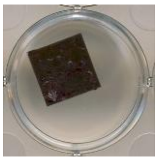

Note: The unmodified composites correspond to the control data previously published in Ref. 25. They are presented here to provide a common reference for comparison with composites modified using different antimicrobial agents.

Table S12. Evaluation of the fungal biofilm formation on the surface of AEH-, HEMA-, and MMA-containing composites using TTC as reaction substrate

**Microorganism / composite**	***C. albicans***	***A. niger***
BPA.DM<br>+AEH	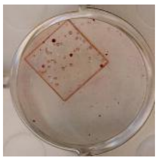	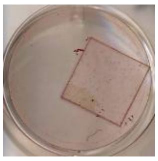
BPA.DM<br>+AEH<br>+BEN	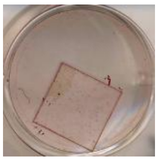	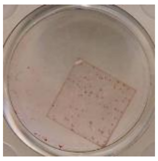
BPA.DM<br>+AEH<br>+$CuSO_4$	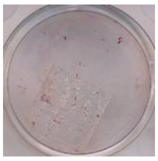	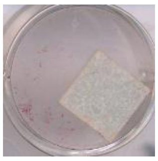
BPA.DM<br>+AEH<br>+Ag	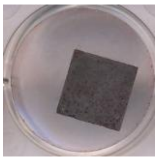	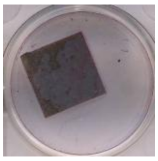
BPA.DM<br>+AEH<br>+ZnO	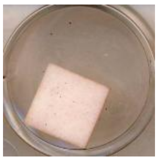	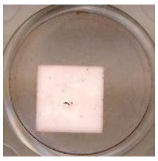
BPA.DM<br>+HEMA	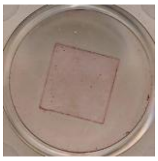	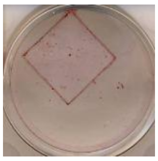
BPA.DM<br>+HEMA<br>+BEN	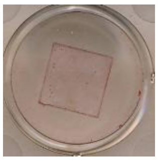	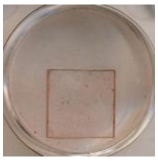
BPA.DM<br>+HEMA<br>+$CuSO_4$	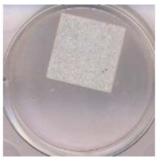	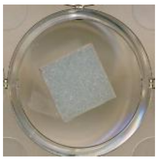
BPA.DM<br>+HEMA<br>+Ag	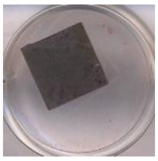	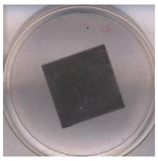
BPA.DM<br>+HEMA<br>+ZnO	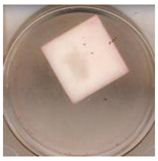	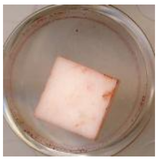

https://doi.org/10.3390/ma19143053

BPA.DM<br>+MMA	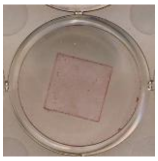	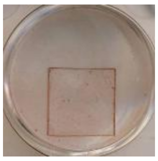
BPA.DM<br>+MMA<br>+BEN	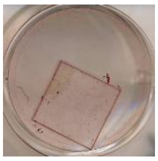	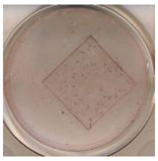
BPA.DM<br>+MMA<br>+$CuSO_4$	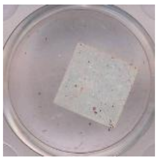	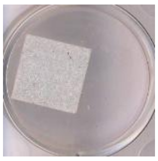
BPA.DM<br>+MMA<br>+Ag	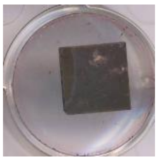	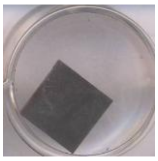
BPA.DM<br>+MMA<br>+ZnO	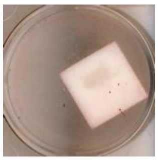	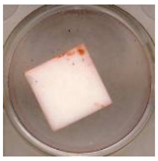
